# Modeling of Zinc Dynamics in the Synaptic Cleft: Implications for Cadherin Mediated Adhesion and Synaptic Plasticity

**DOI:** 10.3389/fnmol.2018.00306

**Published:** 2018-09-04

**Authors:** Christoph Wolf, Agnes Weth, Sebastian Walcher, Christian Lax, Werner Baumgartner

**Affiliations:** ^1^Institute of Medical Biomechatronics, Johannes Kepler University Linz, Linz, Austria; ^2^Lehrstuhl A für Mathematik, RWTH-Aachen University, Aachen, Germany

**Keywords:** cadherins, synaptic plasticity, zinc, analytical model, diffusion, synaptic cleft, long-term potentiation

## Abstract

While the numerous influences of synaptically released zinc on synaptic efficiency during long-term potentiation have been discussed by many authors already, we focused on the possible effect of zinc on cadherins and therefore its contribution to morphological changes in the context of synaptic plasticity. The difficulty with gaining insights into the dynamics of zinc-cadherin interaction is the inability to directly observe it on a suitable timescale. Therefore our approach was to establish an analytical model of the zinc diffusion dynamics in the synaptic cleft and experimentally validate, if the theoretical concentrations at the periphery of the synaptic cleft are sufficient to significantly modulate cadherin-mediated adhesion. Our results emphasize, that synaptically released zinc might have a strong accelerating effect on the morphological changes involved in long-term synaptic plasticity. The approach presented here might also prove useful for investigations on other synaptically released trace metals.

## Introduction

**Figure 1:**
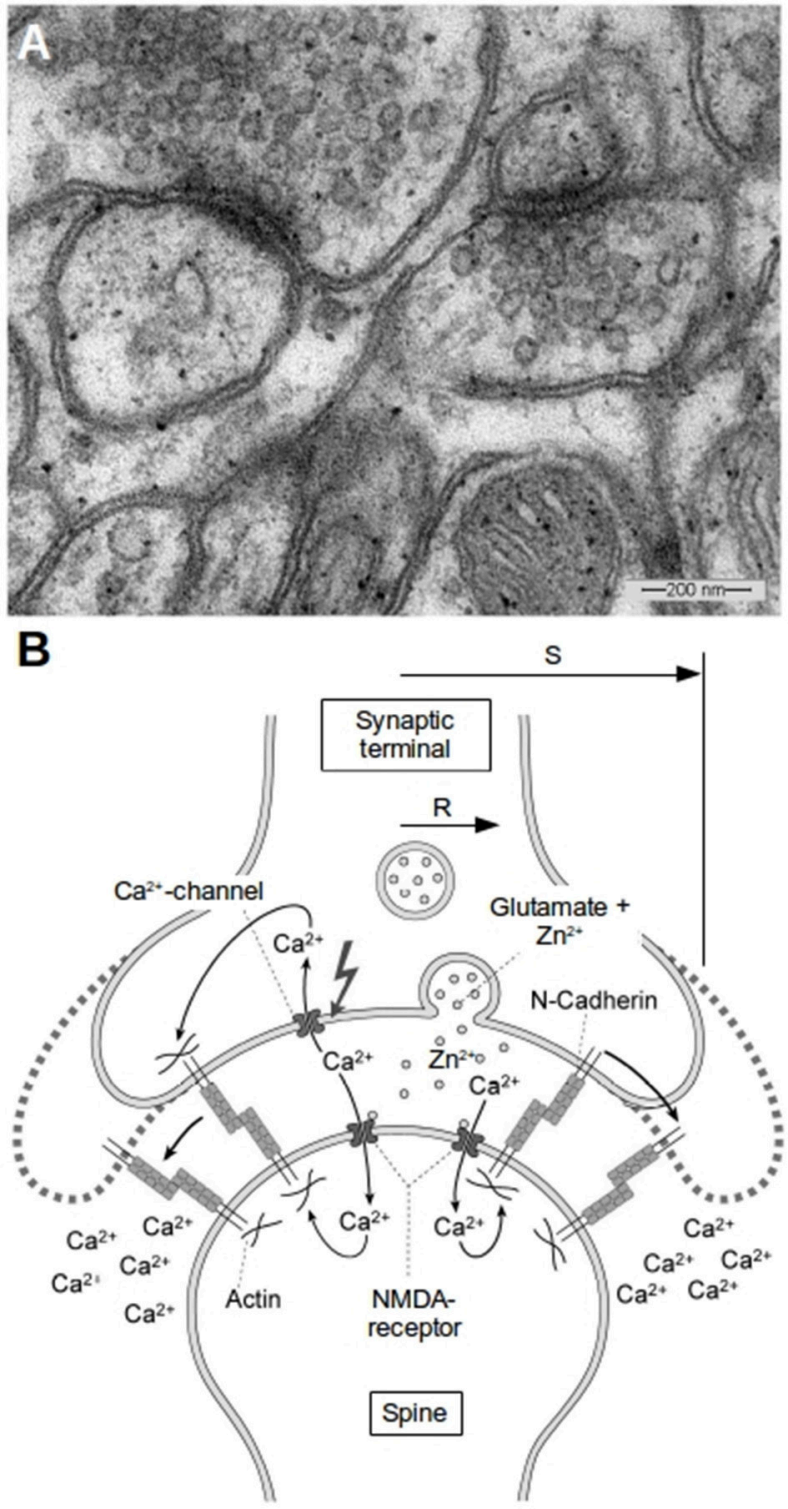
Morphology of glutamatergic synapses. **(A)** A typical EM-image of synapses in the hippocampus of mice. Two synapses can be seen with diameters of the transmissive zones of 200–300 nm and synaptic vesicles in the presynapse which have average diameters of about 30 nm. **(B)** Schematic of a synapse and the proposed mechanism of activity induced growth. The synapse has a transmissive zone (area where vesicle release occurs and where important channels are located) of radius R and an outer radius S. Outside of S we assume the concentrations of ions like Zn^2+^ or Ca^2+^ to be kept constant at background level. The idea of our model is that the increased Zn^2+^-level plays in concert with the increased cytosolic Ca^2+^-level as well as with the reduced extracellular Ca^2+^-level in the cleft to weaken N-cadherin trans-interactions which leads to growth of the synapse.

Zinc is an important trace metal in the brain playing a crucial role in synaptic plasticity, which is the basis for learning and memory. The effects of zinc as well as dysregulation of zinc homeostasis in the nervous system are well established (Huang, 1997; Cole et al., 2000; Frederickson et al., 2000; Lee et al., 2000, 2004; Bancila et al., 2004; Deshpande et al., 2009; Paoletti et al., 2009). Although most of the total zinc in the brain is protein-bound (>80%), free or loosely bound zinc ions (Zn^2+^) can be found pre-dominantly within synaptic vesicles of glutamatergic synapses at concentrations up to 1 mM or more (Frederickson et al., 2000), where it is co-released with glutamate (Assaf and Chung, 1984; Qian and Noebels, 2005; Frederickson et al., 2006). The diameter of synaptic vesicles is ~30–40 nm [Figure 1, (Wenzel et al., 1997)]. In the central nervous system per action potential typically 1–10 (up to 30 in extreme cases) vesicles fuse with the presynaptic membrane and release their content into the synaptic cleft (von Gersdorff and Matthews, 1994; Kandel et al., 2000). In hippocampal synapses the transmitter release probability is typically far below 1, so not every action potential leads to a vesicle fusion and rarely more than one vesicle fuses per action potential (Redman, 1990; Hessler et al., 1993; Ryan and Smith, 1995). Bursts of action potentials can result in very large numbers of vesicle fusions in very short time (Ryan and Smith, 1995). Also continuous mild stimulation mobilizes large numbers of vesicles (Ikeda and Bekkers, 2009), so the bandwidth of possible physiological zinc release rates and concentrations is rather broad and heavily depends on stimulus intensity and duration.

The vesicles are loaded with zinc by the membrane transporter ZnT-3 (Palmiter et al., 1996), which accomplishes a zinc gradient of ~10^8^ against the cytoplasm (Frederickson et al., 2000). In the brain this zinc transporter is expressed exclusively in the synaptic vesicles of the zincergic system (Wenzel et al., 1997; Cole et al., 1999), so that knocking out the SLC30A3 (ZnT-3) gene only eliminates vesicular free zinc (Cole et al., 1999).

Zinc-containing neurons are found mainly in the limbic system and in cerebrocortical regions (Frederickson et al., 2000) but also in the retina, diencephalon, spinal chord and cerebellum, where the zinc is, in contrast to the telencephalon, mostly released in inhibitory synapses (McAllister and Dyck, 2017). Presumably the release of zinc upon synaptic activity is followed by a massive increase in the local Zn^2+^ concentration in the synaptic cleft and subsequently an increase in the postsynaptic intracellular compartments can be observed (Li et al., 2001b). The absence of synaptically released zinc inhibits normal LTP in mossy fiber → CA3 synapses (Lu et al., 2000) and leads, among other memory deficits, to impaired spatial working memory (Sindreu et al., 2011). The memory deficits are not as severe as one would expect, which might be due to the fact, that zinc dependent LTP seems to inhibit the more common NMDA-receptor dependent form of LTP, which might take over large parts of memory function in ZnT-3 knockout mice (Pan et al., 2011). SLC30A3 polymorphisms in humans can lead to ZnT-3 loss of function phenotypes, that were reported to increase seizure susceptibility as well as the risk of schizophrenia (McAllister and Dyck, 2017). Excessive zinc influx however was shown to lead to LTP attenuation in dentate granule cells, which show zinc independent LTP, causing impaired object recognition memory in rats (Suzuki et al., 2015). Besides the numerous modulatory actions of zinc, also metabotropic (GPR39; Nakashima and Dyck, 2009; McAllister and Dyck, 2017) and ionotropic (ZACN; Marger et al., 2014) dedicated zinc receptors were found in neurons. Also NMDA-receptor dependent LTP and LTD is heavily affected by zinc concentrations in the synaptic cleft, as the different subunits of NMDA receptors carry zinc binding sites with different affinities, which allows for a complex zinc modulation of the receptor function, that can be adjusted by subunit composition (Izumi et al., 2006; Nakashima and Dyck, 2009; Vergnano et al., 2014).

In a recent study we proposed the hypothesis that transient local changes of the free extracellular zinc concentration in the synaptic cleft occurring during synaptic activity might affect N-cadherin binding (Heiliger et al., 2015).

Classical cadherins are Ca^2+^-dependent transmembrane glycoproteins associated with adherens junctions of many different cell types (Shapiro et al., 1995; Yap et al., 1997; Steinberg and McNutt, 1999; Angst et al., 2001). In the central nervous system, a large variety of classical cadherins has been reported to be expressed (Redies, 2000; Bekirov et al., 2002) where some are restricted to specific brain regions, whereas others are widely distributed over several regions and different types of synapses. N-cadherin is typically associated with excitatory synapses (Fannon and Colman, 1996; Manabe et al., 2000). Cadherins are known to play a major role during brain development and are key regulators of axonal path finding and specific synapse formation via their homophilic binding activity (Doherty and Walsh, 1996; Redies, 2000; Salinas and Price, 2005; Paradis et al., 2007). Clearly for the reformation of a synapse, homophilic cadherin-interactions have to be temporarily released and reformed in a different way in order to allow synapse growth or shrinkage. Thus an activity dependent binding behavior of cadherins of synapses showing LTP or LTD is vital for learning and memory.

A common theory predicts that due to synaptic activity the Ca^2+^-level in the small synaptic cleft could drop to values at which trans-interaction of N-cadherin molecules is not longer possible. This in combination with the depolymerization of actin due to the elevated Ca^2+^-levels in the cells could lead to diffusion of the cadherin molecules. At lateral positions, i.e. away from the active zone of the synapse, the Ca^2+^-concentrations are normal and here the N-cadherin molecules can reestablish their homophilic interactions. This finally leads to growth of the synapse, especially of the transmissive zone (Marrone and Petit, 2002; Baumgartner et al., 2003, 2013; Matsuzaki et al., 2004; Heupel et al., 2008; Bartelt-Kirbach et al., 2010). A schematic of this model is shown in Figure 1.

In our recent work we could show that elevated Zn^2+^-levels modulate the binding of N-cadherin (Heiliger et al., 2015). The concentration dependency of cadherin-binding inhibition was characterized and the most probable stochiometric ratio was determined to two zinc ions per cadherin molecule. Also zinc was shown to bind directly to N-Cadherin using affinity chromatography. Thus, the interplay between zinc and N-cadherin might provide an additional molecular basis for the structural alterations accompanying plasticity of zincergic excitatory synapses. We could show that addition of 2 μM of zinc, which corresponds to an increase of the free Zn^2+^-concentration of about 400 pM is sufficient to inhibit cadherin binding to control levels (unspecific adhesion found in the absence of Ca^2+^). The question arises whether the dynamics of Zn^2+^-elevations which occur in the synaptic cleft during typical events, that are associated with LTP fits the dynamics of cadherin modulation by zinc.

There are a lot of studies measuring zinc-dynamics in the brain, especially by fluorescence-techniques or advanced spectroscopic methods yielding a lot of valuable information on long term zinc signals and zinc-homeostasis (Li et al., 2001a,b; Varea et al., 2001; Qian and Noebels, 2005). However, the spatial and temporal resolution is not sufficient to resolve single release events in single synapses on a time scale of about 100 ms, as would be necessary to answer our question. Typically LTP in the hippocampus can be induced by bursts of pulses with frequencies of about 100 Hz for below 1 s. Thus we need good estimates of the free Zn^2+^-levels reached during such a burst as well as for the temporal behavior of the Zn^2+^-concentration. As we found no reliable experimental method, we derived a simple analytical model which yields an estimate for the concentration of particles in the synaptic cleft which are either released during synaptic activity or which are taken up during activity.

Having estimates for the behavior of Zn^2+^, we tried to evaluate whether or not a modulation of the N-cadherin binding could take place within the typical times and concentrations we expect. For that purpose we established a fast perfusion and a flash release of Zn^2+^ using photo-sensitive EDTA. The binding activity could be measured using a magnetic system to remove N-cadherin bound super-paramagnetic polystyrene beads from endogenously N-cadherin expressing cells. This showed that in fact the N-cadherin-mediated binding could be modulated within time frames that are of physiological relevance.

## Theory

### General description

To obtain the concentration distribution of a substance of interest in the synaptic cleft the diffusion equation:

(1) $$\begin{array}{r} \frac{\partial c}{\partial t}=D\cdot \nabla^{2}c+f\left(\vec{x},t,c\right) \end{array}$$

has to be solved for appropriate boundary conditions, representing the morphology of synapses, and with a source-function $f\left(\vec{x},t,c\right)$ representing the generation and destruction of free particles at a given position and time. This $f\left(\vec{x},t,c\right)$ also comprises reaction terms like the interaction with a mobile buffer. Thus in principle one has to solve a complete diffusion-reaction scheme including the substance under consideration, the buffer and the complex, i.e., three coupled partial differential equations of the kind of Equation (1). However, if the diffusion of the buffer substance is slow in comparison to the diffusion of the substance under consideration and if the on-rate of the buffering-reaction is high, then a good approximation is to use Equation (1) for the substance under consideration only with a reduced diffusion coefficient (Neher, 1986):

(2) $$\begin{array}{r} \bar{D}=\frac{D}{1+G}\text{ with }G=c_{B}\cdot \frac{k_{on}}{k_{off}} \end{array}$$

With *c*_*B*_ being the concentration of the buffer substance and *k*_*on*_ and *k*_*off*_ describing the on- and off-rate of the buffering reaction.

The general geometry is depicted in Figure 1. The synaptic cleft is represented as rotational symetric and flat. There is an transmissive zone (active zone) of radius R where release of Zn^2+^ (or other particles) occurs. In principle the boundary conditions are mixed boundary conditions with Neumann-conditions at the membrane of the pre- and post-synapse and Dirichlet-boundary conditions at the radial end of the synapse, where the concentration is assumed to be constant. However, it is legitimate to use the source term

(3) $$f(r,t)=\{\begin{array}{c} k||r||\leq R\\ 0\;else \end{array}\}$$

instead of Neumann-boundary-conditions representing the transport between the cleft and the presynapse by either vesicle fusion, channel activity or other transport functions. As the synaptic cleft is thin in comparison to the diameter, we can neglect the concentration changes in *z*-direction and assume the problem to be rotational symmetric (cylinder symmetry).

Without restriction of generality we can apply the initial condition *c*(*t* = 0) = 0. The boundary conditions are

(4) $$\begin{array}{r} c\left(r\geq S\right)=\;0\wedge \;{\left(\frac{\partial c}{\partial r}\right)}_{r=0}=0 \end{array}$$

with *S* denoting the outer diameter of the synapse. Thus the concentration outside of the synapse is constant and we demand the concentration to be continuously differentiable at *r* = 0. Thus outside of radius S we assume to have a large reservoir, corresponding to the extracellular matrix or there is a glia cell, which effectively brings the concentration to 0. Another stationary value is of course also possible and requires only an additive therm in the following equations.

### Stationary solution

As in space *c* depends only on the radial coordinate *r*, one obtains for the stationary case, i.e., if $\frac{\partial c}{\partial t}=0$ the following Poisson-equation:

(5) $$\begin{array}{r} \nabla^{2}c=\frac{1}{r}\frac{\partial }{\partial r}\left(r\frac{\partial c}{\partial r}\right)=-K \end{array}$$

with *K* being a constant which is *k/D* in the transmissive zone, i.e., for *r*<*R* and which is 0 everywhere else. Thus we can solve this ODE for *r*<*R* and for *r* > *R* and we have to choose the integration constants in a way to have the result steady and differentiable at *r* = *R*. The general solution can be obtained by integrating twice leading to:

(6) $$\Rightarrow c=\int \frac{\int r\cdot Kdr+C_{1}}{r}+C_{2}=\frac{-r^{2}\cdot K}{4}-C_{1}\cdot \ln\left(r\right)+C_{2}$$

The integration constants *C*_1_ and *C*_2_ have to be obtained for the two cases *r* ≤ *R* and *r* ≥*R* where both have to yield an equal solution at *r* = *R*. Thus from the demands that *c(r)* is continuously differentiable at all *r*∈[0,S] we obtain

$$\begin{array}{rcl} \Rightarrow c & = & \frac{K}{4}\left(R^{2}-r^{2}\right)+\frac{R^{2}\cdot K}{2}\cdot \ln\left(\frac{S}{R}\right)\forall \;r\leq R\text{ and} \end{array}$$

(7) $$\begin{array}{rcl} c\; & = & \frac{R^{2}\cdot K}{2}\cdot \ln\left(\frac{S}{r}\right)\forall \;r\geq R \end{array}$$

Here *K* = *k/D*. Thus we observe a parabolic decrease of the concentration with *r* in the transmission zone (*r*<*R*) and then a logarithmic decrease outside of the transmission zone.

### Estimation of the time behavior

A general solution of Equation (1) can in principle be found using the separation of variables. Thus we can describe the concentration *c(r,t)* as the product of one time dependent and one space dependent variable as:

(8) $$\begin{array}{r} c\left(r,t\right)=A\left(r\right)\cdot B\left(t\right) \end{array}$$

Thus Equation (1) can be rewritten as:

(9) $$\begin{array}{r} A\left(r\right)\frac{\partial B\left(t\right)}{\partial t}=\frac{D}{r}\frac{\partial }{\partial r}\left[r\frac{\partial A\left(r\right)}{\partial r}\right]+f\left(r,t\right) \end{array}$$

The homogeneous differential equation thus reads:

(10) $$\begin{array}{r} \frac{1}{B\left(t\right)}\frac{\partial B\left(t\right)}{\partial t}=\frac{D}{A\left(r\right)\cdot r}\frac{\partial }{\partial r}\left[r\frac{\partial A\left(r\right)}{\partial r}\right] \end{array}$$

For this equation to be fulfilled for every *r* and *t*, the *r*- and *t*-dependent functions have to fulfill the separated differential equations:

$$\begin{array}{rcl} \frac{1}{B\left(t\right)}\frac{\partial B\left(t\right)}{\partial t} & = & -\lambda \text{ and }\frac{D}{A\left(r\right)\cdot r}\frac{\partial }{\partial r}\left[r\frac{\partial A\left(r\right)}{\partial r}\right]=-\lambda \end{array}$$

(11) $$\begin{array}{r} \text{with}\lambda =\text{constant} \end{array}$$

The minus sign is for simplicity in the further steps. Thus for the time dependent function *B(t)* we obtain:

(12) $$\begin{array}{r} B\left(t\right)=e^{-\lambda t} \end{array}$$

and for the space dependent *A(t)* we obtain:

(13) $$\begin{array}{r} A\left(t\right)=C_{1}\cdot J_{0}\left(\sqrt{\frac{\lambda }{D}}\cdot r\right)+C_{2}\cdot Y_{0}\left(\sqrt{\frac{\lambda }{D}}\cdot r\right) \end{array}$$

with *J*_0_ being the Bessel function of the first kind of order 0 and *Y*_0_ being the modified Bessel function of the first kind of order 0. For physical reasons we demand that the derivative of *C(t,r)* at *r* = *0* has to be zero thus the constants *C*_2_ have to be zero as the modified Bessel function of the first kind tends to infinity at the origin. Thus we obtain:

(14) $$\begin{array}{r} A\left(r\right)=C_{1}\cdot J_{0}\left(\sqrt{\frac{\lambda }{D}}\cdot r\right) \end{array}$$

A general solution for *B(t)* and *A(r)* is hard to obtain as one has to find appropriate Green-functions for the given geometry. The complete solution for the inhomogeneous PDE is even more complicated. However, from the above formula we can derive the slowest time constant of the system. We demand that:

(15) $$\begin{array}{r} A\left(r=S\right)=C_{1}\cdot J_{0}\left(\sqrt{\frac{\lambda }{D}}\cdot S\right)=0 \end{array}$$

according to the boundary conditions. Thus in order to obtain any non trivial solution, i.e., for *C*_1_≠0 we obtain:

(16) $$\begin{array}{r} \sqrt{\frac{\lambda }{D}}\cdot S=R_{J0} \end{array}$$

with *R*_*J*__0_ being a root of the Bessel function *J*_0_. These roots can not be obtained analytically, however, the first one is ~ 2.4048. Thus we can approximate the corresponding constant λ, which is the reciprocal of the slowest time constant occurring in the system to be

(17) $$\begin{array}{r} \lambda_{min}=\frac{1}{\tau_{max}}=D\cdot {\left(\frac{2.4048}{S}\right)}^{2} \end{array}$$

For the higher order roots of the Bessel function we obtain faster time constants. Thus the stationary (asymptotic) solution in the cleft given by Equation (7) will be reached exponentially with the time constant τ_*max*_. If major changes in *f(r,t)* occur only with time constants significantly larger than τ_*max*_, one can roughly estimate the time and space dependent concentration to be

$$\begin{array}{rcl} c\left(r,t\right) & \approx & c_{stat}\left(r\right)\cdot \left[1-\text{exp}\left(\frac{-t}{\tau_{max}}\right)\right]\text{or} \end{array}$$

(18) $$\begin{array}{rcl} c\left(r,t\right) & \approx & c_{stat}\left(r\right)\cdot \exp\left(\frac{-t}{\tau_{max}}\right) \end{array}$$

with *c*_*stat*_*(r)* being the stationary solution given by Equation (7). The first equation describes the changes from the initial conditions for which *f(r,t)* has been zero for long time and which is changed to Equation (3) at time 0. The second equation describes the return to the initial conditions after the stimulation has ended, i.e., when *f(r,t)* became zero again.

### Numerical simulation

To evaluate the analytical model we implemented a finite volume simulation similar to the method developed recently (Ahl et al., 2011). It was adapted only in the sense as we assume rotational symmetry and convectional therms could be neglected. For the simulation we assumed a time behavior of the release as follows: At a frequency of 100 Hz action potentials are assumed which lead to a release for 5 ms. The other parameters are assumed to be identical to the analytical model. In order to estimate the influence of zinc uptake due to ATPases in the pre- and postsynaptic membrane we also allowed a concentration dependent transmembrane transport.

## Materials and methods

### Cell culture and protein purification

PC12 rat pheochromocytoma cells (ECACC 88022401), endogenously expressing N-cadherin, were cultured in DMEM medium containing 5% FCS and 10% horse serum (both from Biochrom, Berlin,Germany), 2 mM glutamine, 50 U ml^−1^ penicillin-G and 50 U ml^−1^ streptomycin (all from Sigma) at 37°C in a humidified atmosphere with 5% CO_2_.

N-cadherin-Fc fusion protein (mouse N-cadherin extracellular domain fused to Fc-portion of human IgG1, called N-cad-Fc in the following) was generated as described recently (Heiliger et al., 2015). Chinese hamster ovary cells stable transfected with the N-cadherin-Fc construct secreted the N-cad-Fc protein. This protein was purified from the culture supernatants by affinity chromatography using protein A agarose (Merck, Darmstadt, Germany).

### Coating of polystyrene beads

N-cad-Fc was coupled to protein A-coated superparamagnetic polystyrene microbeads (Dynabeads, diameter 2.8 μm, Dynal, Oslo) as described (Heiliger et al., 2015). Briefly, after vortexing 10 ml bead suspension were washed 3 times in 100 ml of buffer A (100 mM Na-phosphate, pH 8.1) by sedimenting the beads via a magnet and reuptake in buffer A. The washed beads were suspended in 100 ml buffer A containing 10 mg of cadherin-Fc protein and allowed to react for 30 min at room temperature under permanent slow overhead rotation. After washing 3 times in 100 ml buffer B (200 mM triethanol- amine, pH 9.0) beads were incubated for 45 min in 100 ml buffer B containing 0.54 mg dimethyl pimelimidate ∙ 2HCl (DMP, Pierce,Rockford, USA) at RT. Free DMP was blocked by two washes for 30 min at 37°C in 100 ml 100 mM Tris pH 8.0. Finally the beads were washed 3 times in Hanks Balanced Salt Solution (HBSS, Gibco, Karlsruhe). Beads were stored for up to 7 days in HBSS containing 0.1% BSA and 0.02% sodium azide at 4°C at slow overhead rotation to avoid sedimentation.

### Magnetic tweezer experiments

The electromagnet setup, which was used as a “magnetic tweezer,” consists of an acrylic post, that was machined on a lathe to carry a coil on the outside and a thin iron rod on the inside, which protrudes the end of the pole. The coil was connected to a regulated power source, so that it generates an electromagnetic field, which is extended by the iron rod. The acrylic pole was mounted vertically, with the iron rod facing down, on an inverse microscope. As the acrylic rod was replacing the light source of the microscope, a light source had to be integrated into the electromagnet. This was done by polishing the ends of the acrylic pole and mounting a high output LED-chip on the top end. In this setup the light shines through the acrylic rod and illuminates the sample on the microscope. As the iron rod is situated in the center of the light path, its shadow darkens the image on the microscope when it is lowered too deep. This is not only a safety feature to avoid damaging the subject, but it also allows dark-field microscopy, which theoretically offers better contrast on the beads. Unfortunately we were not able to take advantage of this option, because in the dark-field it was impossible to reliably distinguish between beads, that were attached to the upper surface of a single cell and those, that were rather incarcerated between two cells, as only the former were incorporated in our examination.

The lower end of the post was machined to fit a standard 35 mm well on a polystyrene multiwell plate with some room for horizontal movement. As the post was mounted on a frame with a vertical positioning mechanism, the applying of the beads could be performed without demounting the electromagnet. Afterwards the magnet was lowered down until it touched the surface of the medium.

The experiment was carried out in polystyrene petri dishes (Greiner, Frickenhausen) coated with poly-L-lysin (Sigma-Aldrich, Vienna, Austria) and filled with cell culture medium. Five petridishes were used with three measurements conducted on different spots of each petridish. The results were averaged and their standard deviation was used for the error bars in the corresponding figures. The electromagnet was driven with 200 mA, which has empirically proven (using EGTA to remove calcium and therefore cancel cadherin-mediated adhesion) to provide a reasonable magnetic force to remove unbound beads. As the coil of the magnet was handwound, the magnetic flux can only be roughly estimated to 5 mWb.

**Figure 2:**
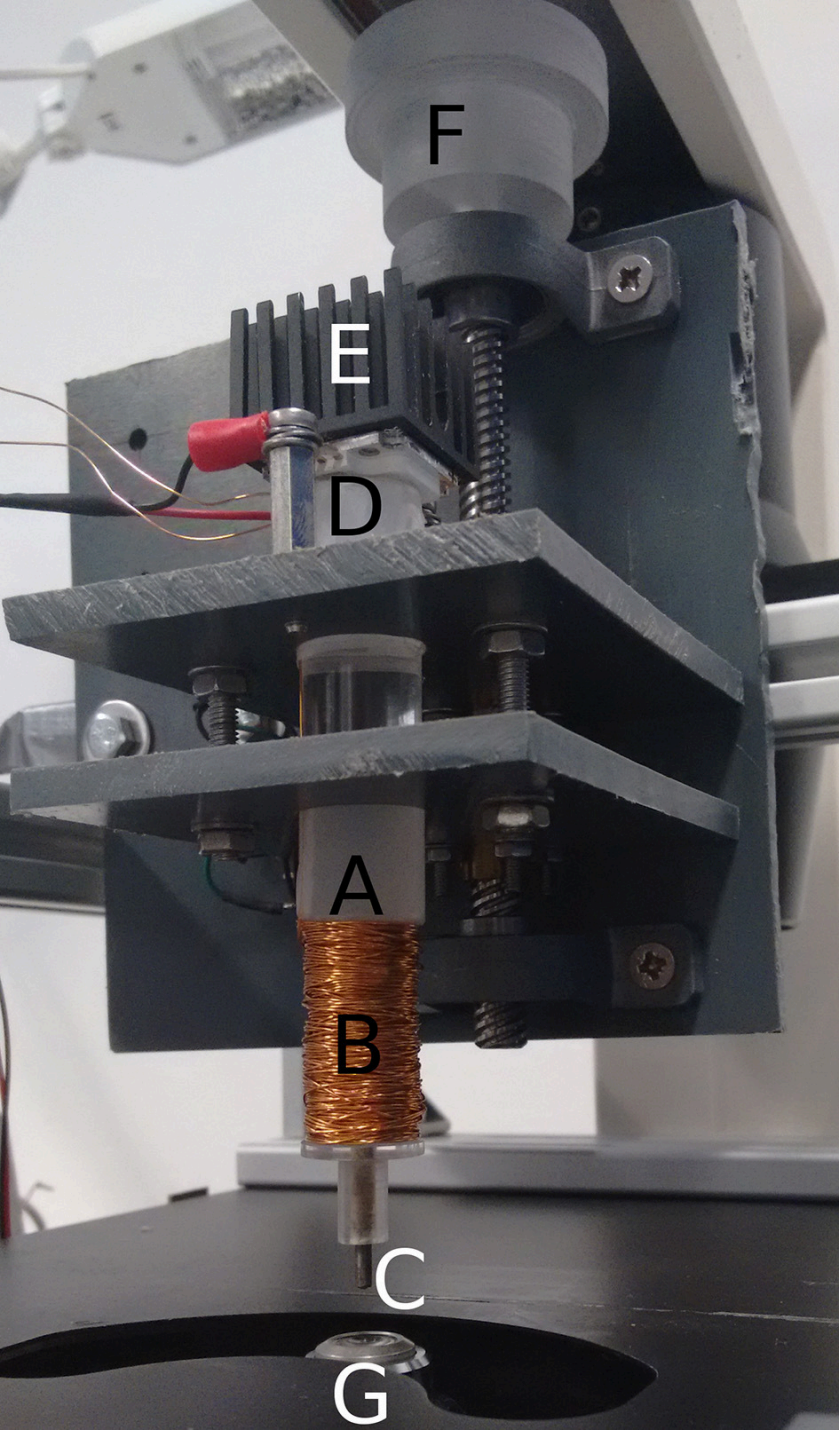
Setup of the illuminated electromagnet. Acrylic pole **(A)**, copper coil **(B)**, iron rod **(C)**, LED-chip **(D)**, heatsink **(E)**, vertical positioning mechanism **(F)**, objective of the microscope **(G)**.

The actual setup is shown in Figure 2.

### Photometric determination of free zinc and zinc release by DMNP-EDTA

The concentration of free zinc in presence of DMNP-EDTA, before and after UV-light induced degradation of the complex, was assessed using the high affinity fluorescent probe FluoZin-3. Due to the high affinity and high fluorescence output of FluoZin-3, the concentrations of zinc and DMNP-EDTA in this experiment had to be reduced by a factor of 100 compared to the magnetic tweezer experiment.

To also assess the effect of the calcium in the cell culture medium, as DMNP-EDTA is not specific for zinc and even has a higher affinity for calcium, the zinc release experiment was performed with two concentrations of calcium additionally. As the calcium concentration as well as the UV-light treatment affect the fluorescence of FluoZin-3, calibration curves had to be taken separately for every single measurement. The fluorescence curve of FluoZin-3 as a function of free zinc concentration has the form:

(19) $$\begin{array}{r} F=\frac{a}{b+c\cdot e^{-d\left(\left[Zn\right]-f\right)}}+t \end{array}$$

Where *F* is the fluorescence intensity, [*Zn*] is the free zinc concentration and the parameters *a, b, c, d, f*, and *t* are determined by a genetic algorithm (DEPS Evolutionary Algorithm, as part of the LibreOffice Calc extension “nlpsolver” 0.9) from a calibration series for each measurement, so that calcium concentration and light exposure are always properly taken into account.

To calculate the free zinc concentration from the measured fluorescence intensity, the function can be inverted for [*Zn*]:

(20) $$\begin{array}{r} \left[Zn\right]=\frac{\ln\left(\frac{\frac{a}{F-t}-b}{c}\right)}{-d}+f \end{array}$$

The fluorescence intensity was measured in a plate reader (Promega Glo-Max® Multi) using a standard 96-well plate for calibration series and samples. The UV-treatment was conducted using a portable UV-chamber (Sina DR 302). The Microwell-Plate was loaded with three rows with calcium concentrations of 0, 200 μM, and 2 mM. The columns contained Zn^2+^ concentrations ranging from 0 over 50 pM to 250 nM for calibration and one well per row with 1 μM Zn^2+^ preloaded DMNP-EDTA.

All loaded wells were stained with 3 μM FluoZin-3.

As preliminary experiments have shown, with 250 nM zinc, the fluorescence signal is already in the upper asymptotic part of the calibration function, but the actually released concentration of zinc from the 1 μM DMNP-EDTA is far below this value and can rather be located in the semi-linear range of the function. For the free zinc concentration before UV-treatment, a time sequence of 5 measurements spread over 10 min was taken, to make sure, that the zinc is not continuously released from the complex. As the zinc release depends on the duration of UV-treatment and the fluorescent probe is bleached by the broadspectrum UV light, two different UV-treatment durations were applied to each sample sequentially, with recovery periods afterwards, for the dye to partially restore it's fluorescent signal and therefore increase the accuracy of the reading.

## Results

**Figure 3:**
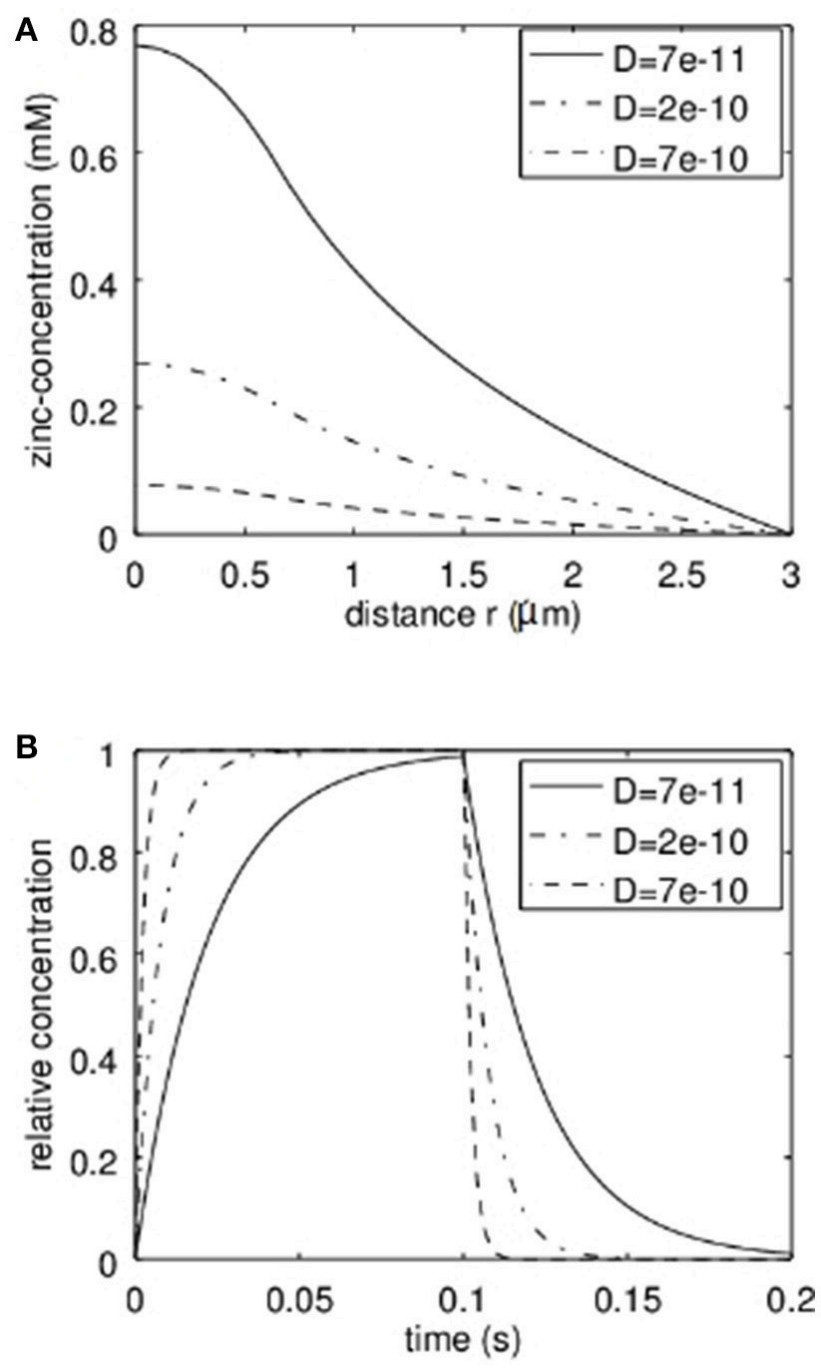
Analytical approximation of the total Zn^2+^-concentration in the synaptic cleft (Including bound and therefore non-free zinc ions). As discussed, the actual “free” zinc concentration is much lower. In **(A)** the stationary concentration of Zn^2+^ in dependence on the radial position r can be seen. The concentration is depicted for different values of the effective diffusion coefficient. In **(B)** the time behavior based on our first order approximation is given for different values of the effective diffusion coefficient D. This time course multiplied with the stationary concentration at a given radius r (from **A**) yields the approximation of the time course of the concentration at a given position.

First the analytical model was applied to estimate the concentrations reached in the synaptic cleft. In Figure 3A the stationary solution according to equation 7 can be seen. Here a radius of the transmission zone (active zone) of *R* = 300 nm was assumed. The outer synapse radius S = 3 μm which means that we assume the concentration for *r* > 3 μm to be zero either due to a big buffering reservoir like the extracellular matrix or due to glia cells effectively removing the released Zn^2+^. The results are shown for different values of the effective diffusion coefficients. The diffusion coefficient of free Zn^2+^ in the absence of zinc binding proteins and diffusion hindering structures like a glycocalix in an aqueous solution would be 7·10^−10^m2/s (dashed line in Figure 3). However, we can assume that in the synaptic cleft the diffusion is slowed down. According to (Hodgkin and Keynes, 1957; Nasi and Tillotson, 1985; Nielsen et al., 2004) at least a factor of 3 should be assumed. That is why diffusion coefficients of 2·10^−10^m2/s and 7·10^−11^m2/2 are also shown in Figure 3. Clearly Figure 3A shows the parabolic initial decrease for *r*<*R* and then the logarithmic behavior. The dependence on the diffusion coefficient can be clearly seen. It has to be emphasized that in Figure 3A the total concentration of zinc-ions due to the release is shown. The concentration of free ions is of course much lower. The time behavior of the relative zinc-concentration is shown in Figure 3B according to equation 18. Here c_stat_ is set to 1 to show the relative behavior. Clearly if the diffusion coefficient decreases, the time until the stationary solution is reached increases. However, even for a diffusion coefficient 10 times smaller than for the free diffusion in aqueous solution the stationary Zn^2+^-level is reached within times that are shorter than typical stimulation bursts (e.g., 1 s) used for LTP-induction.

**Figure 4:**
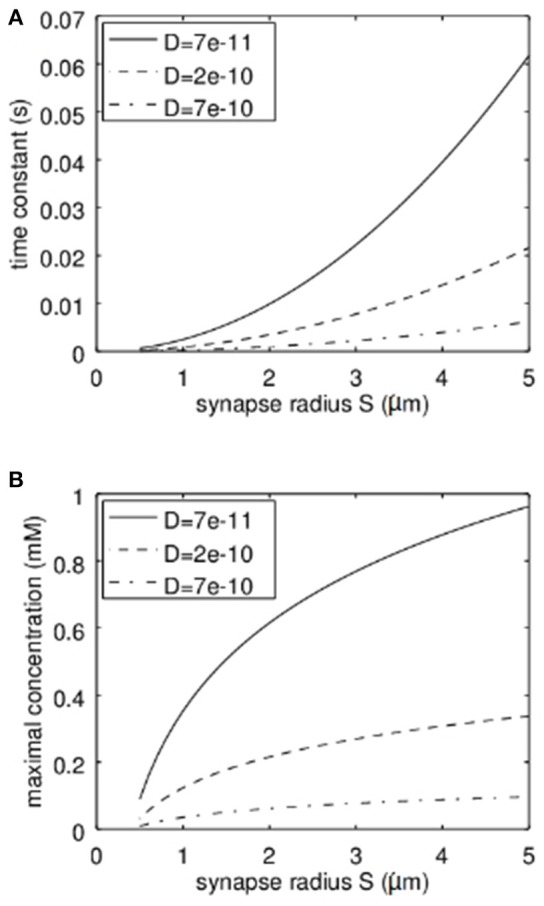
Dependence of the time constant of Zn^2+^-changes and the maximum concentration of Zn^2+^ in dependence on the synapse radius S and the effective diffusion coefficient. **(A)** Here the quadratic increase of the time constant can be seen. The dependence on the diffusion coefficient is linear. **(B)** Here the logarithmic dependence of the maximal concentration on the synapse radius S can be recognized while also here the dependence on the diffusion coefficient is linear.

It is clear that the diffusion coefficient influences the stationary solution as well as the time constant. However, another important parameter is the synapse geometry. In Figure 4 the dependence of the time constant according to equation 17 and the maximal concentration (stationary solution at *r* = 0) on the synapse radius *S* are shown. Not surprisingly the larger the synapse the higher the time constant and the higher the maximal Zn^2+^-concentration. For typical settings the time constants are clearly below 1 s. Again it has to be emphasized that the concentrations shown are the total concentrations and not the concentrations of free zinc-ions.

**Figure 5:**
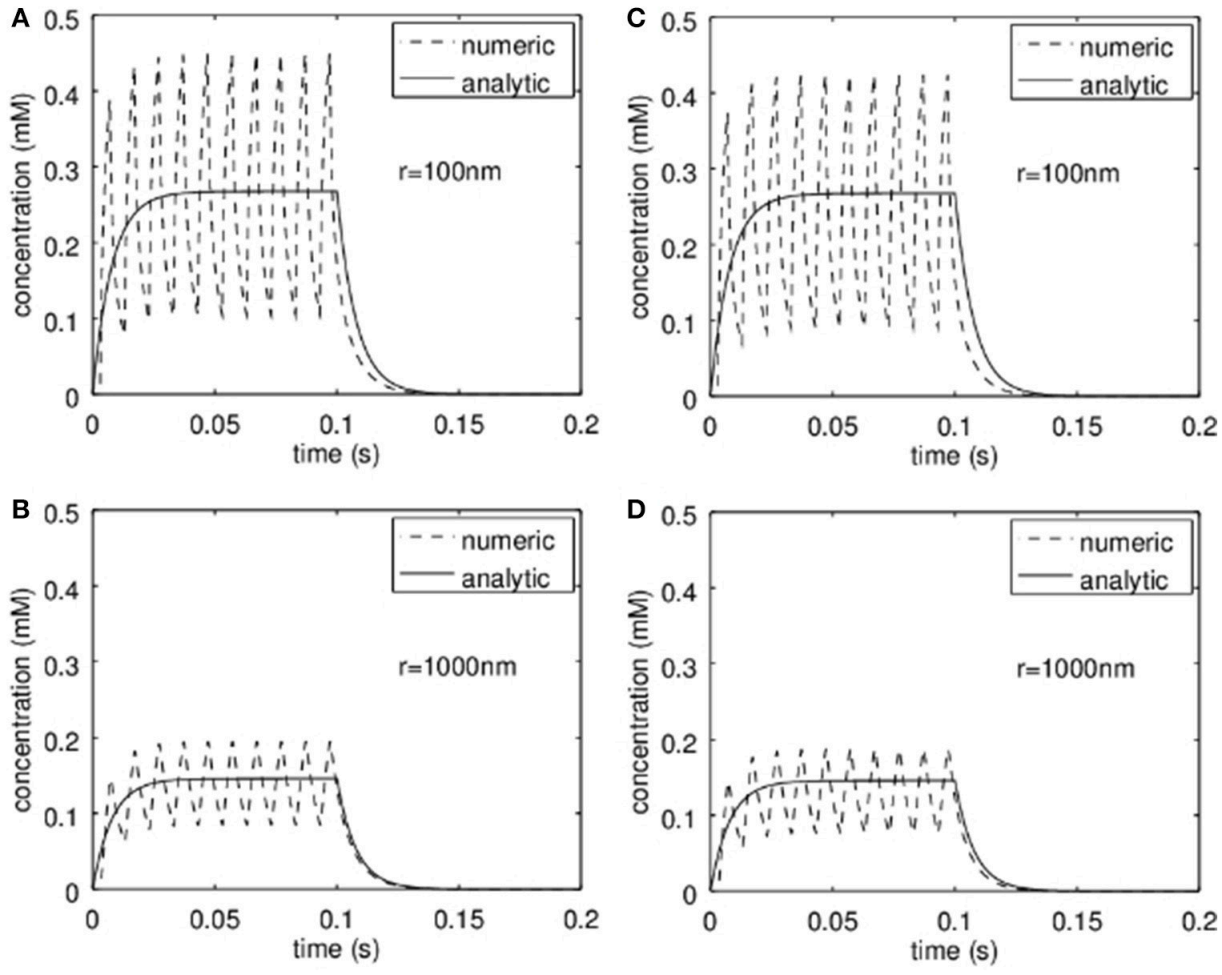
Comparison of the analytic approximation with a finite-volume simulation. **(A,B)** show the time course assuming no active take up of zinc by the pre- and post-synapse respectively at a distance *r* = 100 nm **(A)** and *r* = 1 μm **(B)**. This corresponds to the assumption used for the derivation of the analytical model. In **(C,D)** the analytical solution (identical to that in **A,B**) is compared to a simulation where active take up of zinc is simulated. Clearly at physiological transport rates the concentrations are slightly lower but do not deviate much from the analytical approximation.

For the analytic approximation we assumed to have a constant release of zinc during the stimulation period (e.g., stimulation with 100 Hz for up to 1 s; Lu et al., 2000; Li et al., 2001a,b; Kay, 2003) and furthermore we neglected the zinc-uptake by the neurons themselves. In order to see what happens if the release is intermittent and if the neurons can remove zinc from the synaptic cleft at physiological rates, the analytical solutions were compared to numerical simulations. This is shown in Figure 5. In Figures 5A,B the results are shown if the neurons do not take up zinc in the synaptic cleft, while in Figures 5C,D a rate for the uptake of 3·10^−6^mol/m2/s was assumed. The analytical approximation (solid line) and the numerical simulations (dashed lines) are shown at a radius *r* = 100 nm (Figures 5A,C) and for *r* = 1 μm (Figure 5B). Here S was assumed to be 3 μm and *R* = 500 nm. The diffusion coefficient was set to 2·10^−10^m2/s. Obviously the analytical solution approximates the numerical results quite well. The rather slow uptake yields only negligible deviations as can be seen in Figures 5C,D.

Although our model is only a crude estimation of the real situation where a more complicated geometry is given and where additional zinc-binding molecules change the dynamics, we can learn quite a bit from the analytical solution. We would, even under unfavorable conditions, expect the concentration of zinc in the synaptic cleft to rise within some hundred milliseconds to values of >100 μm. This raises the question, if N-cadherin can respond within such a time- and concentration frame.

**Figure 6:**
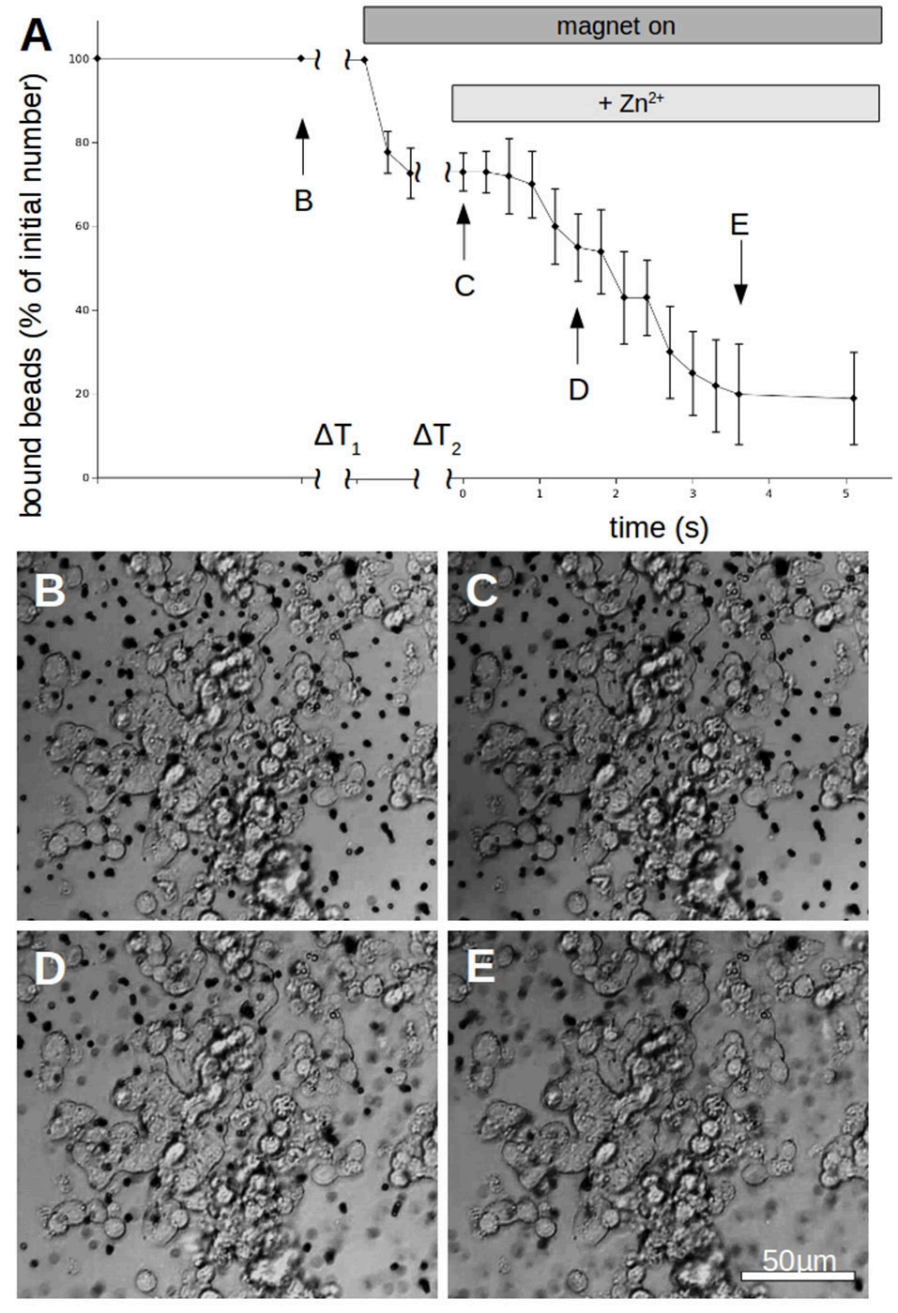
Time behavior of N-cadherin mediated adhesion of N-cadherin coated super-paramagnetic polystyrol-beads on PC12-cells. **(A)** After allowing to settle for a time ΔT_1_ (typically 3 min) the magnet is turned on which removes beads which are not tightly attached. After a time ΔT_2_ (typically 5 s) free zinc is perfused onto the cells to a final total concentration of 100 μM. The percentage of beads resisting removal by the magnet (normalized to the initial number) clearly drops within 4 s. The curve shows for each time value the average numbers of 5 independent trials (different petri dishes). In each petri dish 3 different positions were spotted with typically >50 beads per field of view. Only beads which are clearly on PC12-cells were counted while beads which are in between cells were omitted from the analysis. **(B–E)** Snapshots of the cells with bound beads during one measurement at the times indicated in **(A)**.

**Figure 7:**
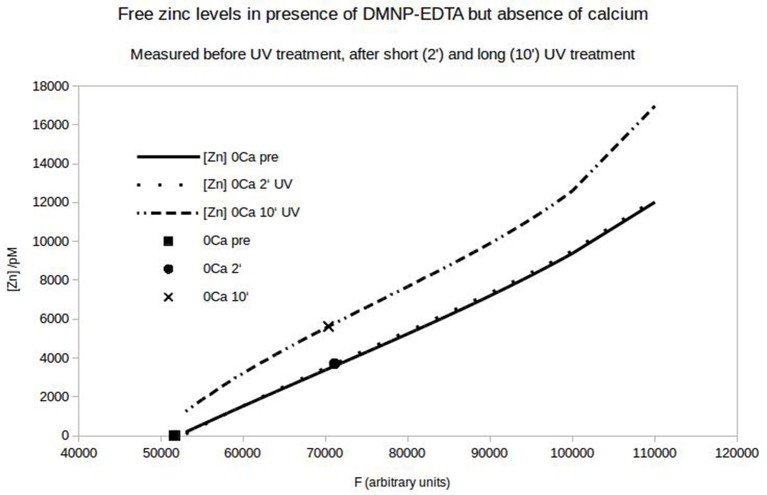
Free zinc-concentration determined by FluoZin-fluorescence. Under control condition a semi-linear dependence of the fluorescence on the added zinc can be seen (solid line). If 1 μM zinc is loaded on DMNP-EDTA, the fluorescence stays at the zero point of the calibration line (square). After UV-exposure (2 or 10 min) the fluorescence increases (circle and x) relative to the matching calibration lines (Light exposure affects calibration).

To quantify the N-cadherin mediated adhesion in dependence on the added zinc, we used N-cadherin-coated super-paramagnetic polystyrene beads. These were allowed to settle on endogeneously N-cadherin-expressing PC12-cells. This was already established by our group and we succeeded in quantifying N-cadherin mediated adhesion by probing the beads with a laser tweezer. However, this technique is very precise but rather slow and would not allow to obtain the time-resolution needed. Thus we replaced the laser by an electro-magnet which removed all non-adhering beads from a large area. The beads and the cells were observed with a microscope and the behavior was recorded with a camera at a rate of 10 frames/s. After the measurement we analyzed the frames by counting the number of attached beads on cells. In Figure 6 the time behavior of the percentage of bound beads averaged over *n* = 5 independent trials is shown. The beads were allowed to settle for a time ΔT_1_ (typically 3 min). Then the magnet was turned on, removing all beads which are not tightly attached. After a time ΔT_2_ (typically 5 s) free zinc was perfused onto the cells to a final total concentration of 100 μM. The pipette was supported by a home built rack in order to keep the distance to the beads in the field of view constant. The percentage of beads resisting removal by the magnet (normalized to the initial number) clearly drops within several seconds. Compared to the 5 experiments we did without adding zinc, yielding little to no bead detachment within the same time frame, the effect of the zinc is highly significant (Wilcoxon-rank-test delivering W = 15, which corresponds to an error probability of α = 5,32^*^10^−4^). Of course the perfusion is cumbersome as we will not obtain a homogeneous increase of the free zinc immediately. Thus we searched for an alternative approach to quickly increase the free zinc-concentration. There is no good commercially available photolabile chelator for zinc. However, we tried DMNP-EDTA which we found to have at least an acceptable performance to show that the time constants are feasible. In Figure 7 a photometric measurement of the free zinc-concentration is shown. The quantification was done by measuring the fluorescence of the zinc-dependent dye FluoZin-3. All samples contained 3 μM of FluoZin-3. If increasing amounts of zinc were added to the samples, a semi-linear increase of the fluorescence could be observed (solid line). However, if 1 μM of zinc was preloaded to DMNP-EDTA (1:1), free zinc was too low to measure (square). After exposure to UV-light, some of the caged zinc is released (circle and x). As the UV-exposure affects the fluorescent dye, the measurement had to be re-calibrated (dotted and dash-dotted lines). The whole procedure was repeated in presence of 0.2 mM, and 2 mM of calcium, to make sure, that the calcium in the cell culture medium does not interfere with the zinc caging too much. The cell culture medium contains 2 mM Ca, so the 0.2 mM sample matches the scaling factor of the zinc concentration. In presence of calcium a very small amount of free zinc could be measured (~ 30 pM or 0.003%), but the signal remained constant over a time course of 10 min, so that no lingering exchange of calcium and zinc at the chelator is to be expected. The fraction of actually released zinc is rather small, due to the high stability of the molecule and the low energy of the UV-source. In the magnetic tweezer experiment a more powerful light-source was used, but that is incompatible with the FluoZin. Also a much higher release quota was obtained using 100 times higher concentrations of zinc loaded DMNP-EDTA, but in this case the FluoZin-3 concentration had to be 1,000 times higher (3 mM) to keep the obtained readouts in the linear range of the calibration function. As the affinity of FluoZin-3 to zinc is very high (K_D_~15 nM), it is unknown to what degree these results were influenced by the competition of the fluorescent indicator with the chelator.

**Figure 8:**
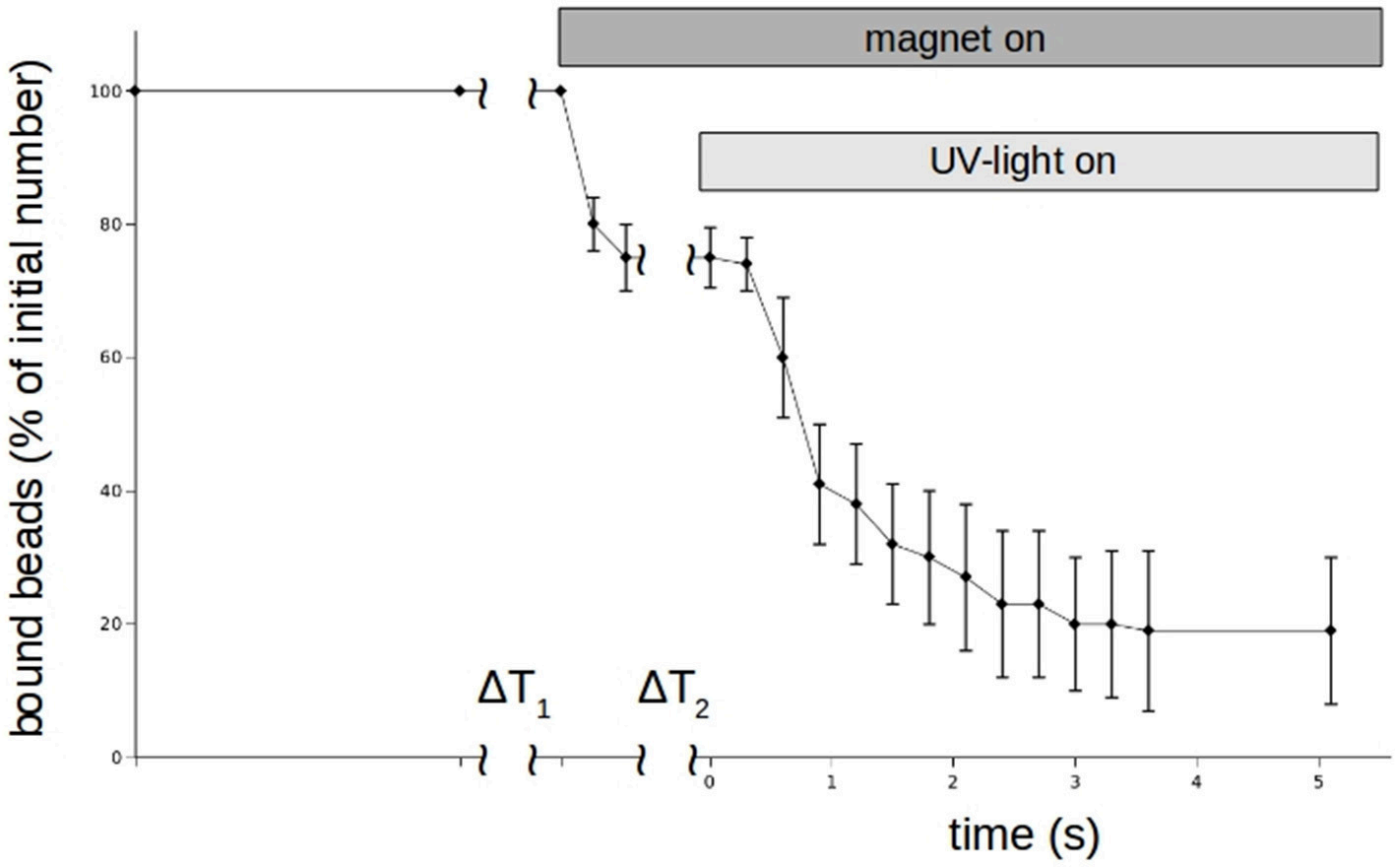
Time behavior of N-cadherin mediated adhesion of N-cadherin coated super-paramagnetic polystyrol-beads on PC12-cells in the presence of 100 μm Zn^2+^-saturated DNMP-EDTA. Like in the experiments shown in Figure 5, beads were allowing to settle for ΔT_1_. Then the magnet is turned on removing loose beads. After a time ΔT_2_ a UV-lamp is turned on which photolyses the DNMP-EDTA. The percentage of beads resisting removal by the magnet (normalized to the initial number) clearly drops even faster than in the case of perfusion. The curve shows for each time value the average numbers of 5 independent trials. Also here we counted only beads which are clearly on and not in between PC12-cells.

Although DMNP-EDTA is not a good photolabile chelator for zinc, we could use it to show that zinc can rapidly modulate N-cadherin mediated adhesion. Again the magnet-setup was used. The results for *n* = 3 independent measurements are shown in Figure 8. Beads were allowed to settle again for about 3 min. Then zinc-loaded DMNP-EDTA was added and allowed to mix with the medium for about 30 s. As Calcium can compete with the zinc, we had to keep the incubation times low. Then the magnet was turned on to remove all beads not tightly bound to the cells. Then the UV-lamp was turned on by opening a shutter to photolyse the DMNP-EDTA. Within 3 s the beads could be removed.

## Discussion

Learning and memory, especially long-term potentiation (LTP), but also long-term depression (LTD), are associated with morphological changes of synapses. For such changes to occur it is necessary that established cadherin mediated adhesive contacts are broken and reestablished in dependence on the synaptic activity. In case of LTD a reversible shrinkage of spines can be observed (Zhou et al., 2004), which probably requires a large amount of cadherin reorganization, as well as synaptic growth does. It is assumed that the local depletion of Ca^2+^ is, at least in part, causally involved in this process (Baumgartner et al., 2003; Heupel et al., 2008). Also local activity dependent changes of the pH-value (Baumgartner et al., 2013) and of the Zn^2+^-concentration (Heiliger et al., 2015) were proposed in the past to play an important role in the modulation of N-cadherin mediated adhesion. Good evidences were provided for this. However, all measurements directly showing that calcium, pH or zinc can modulate cadherin binding were done under static conditions. If the dynamics of the reactions allow such a mechanism was not clear.

In order to get some insights into the dynamics of the dependency of N-cadherin binding on the presence of extracellular substances like Zn^2+^ we developed a simple analytical model for the stationary concentration of released (or uptaken, which corresponds to a negative release) molecules. Furthermore we derived an approximation for the time course of the concentration.

Although simple, the model can help to quickly learn about the influence of different parameters like synaptic morphology and diffusion coefficient on the concentration behavior of different substances in the synaptic cleft. Thus besides helping to estimate the Zn^2+^-response of synaptic activity, the model could prove useful in different fields of the investigation of synapses. For example the spatial and temporal behavior of other ions like Ca^2+^ or Cu^2+^ could be investigated.

The model predicts, that within typical stimulation times the released zinc-concentration can rise up to some hundred μM. It can not be overemphasized that the actual free zinc-concentration is definitely much lower but also much harder to determine, as the full kinematic data of all zinc-binding molecules and their concentrations would need to be known. Under stationary conditions it is known that in our cell culture medium used for the PC12-cells an addition of zinc up to 2 μM only yields an increase of the free zinc of about 400 pM (Heiliger et al., 2015). How fast these reactions take place is not known. It is also not known if the situation in cell culture medium is similar to the conditions within the synaptic cleft.

However, assuming the concentration and time courses to be of the right order of magnitude, we performed some experiments trying to show that in fact fast but high transients (up to 100 μM) of zinc can modulate N-cadherin mediated adhesion. As a first attempt we used a fast perfusion of N-cadherin-coated beads adhering to N-cadherin expressing PC12-cells. To have a fast readout we had to use a magnet to probe the adhesive strength of the bead-cell-interaction. We are aware that this is a rather crude method, but it is a fast and simple way. It was found that in fact the beads can be released from the cells within some seconds.

Our findings are in accordance with previous work from other groups, especially Vergnano et al. (2014), that did show, that the number of zinc ions per vesicle is up to approximately 125 (with concentrations ranging from 1–5 mM, although we used a more conservative estimate of ~28 ions, corresponding to 1–1.5 mM), as they found the zinc- glutamate ratio within the vesicles to be 1:20. The actual free zinc concentration was not found to peak over 10 μM under physiological conditions, even after high frequency stimulation (HFS), which renders interactions with low affinity binding sites unlikely (Vergnano et al., 2014). The release probabilities for a single release site synapse, like the Shaffer collateral → CA1 synapse range from 0.3 (baseline) to 0.5 (after HFS) (Vergnano et al., 2014). Our results state, that a synapse of 300 nm active zone radius and 40 nm cleft width would bear 500–6000 additional Zn^2+^ ions in the synaptic cleft after 1 s of 100 Hz stimulation. We calculated this with a constant release probability of 0.5. It is important to keep in mind, that not all the released zinc ends up as free zinc, as the zinc elimination mechanisms within the synaptic cleft will make a lot of the total released zinc unavailable right away. How much of the released zinc is actually available to a particular interaction site depends on the kinetics and can therefore not be expressed with a general reduction factor. The effectiveness of the synaptic zinc elimination mechanisms is impressively demonstrated by the quite large difference between baseline synaptic zinc levels and the exterior zinc concentration within the cerebrospinal fluid and extracellular medium, which is significantly higher (Vergnano et al., 2014).

As an alternative approach to get rid of potential artifacts due to the perfusion, we tried to release zinc by photolysis. As no good commercial photolabile chelator particularly for zinc is available, we used DMNP-EDTA. We found by photometric characterisation that in fact this chelator is a rather weak cage for flash photolysis of zinc. However, when working fast in order to avoid slow release due to the competition of zinc with calcium and magnesium, it is possible to set free significant amounts of zinc by a UV-flash. Unfortunately a detailed quantification is hardly possible due to the weak and not completely resolved kinetics of DMNP-EDTA interaction with zinc.

However, the results obtained so far indicate that a fast modulation of N-cadherin binding by released zinc in the range of physiological parameters is possible and likely to occur. More work will be necessary in the future to obtain more precisely as to how zinc influences the synaptic plasticity by extracellular actions.

## Author contributions

The practical experiments were carried out by WB, CW, and AW. AW was in charge of the cell culture, CW engineered the magnetic tweezer setup. The analytical model was designed by WB, SW, and CL, the numerical simulation was carried out by WB. The writing was done by CW and WB.

### Conflict of interest statement

The authors declare that the research was conducted in the absence of any commercial or financial relationships that could be construed as a potential conflict of interest.
